# Organised food crime: an analysis of the involvements of organised crime groups in the food sector in England and Italy

**DOI:** 10.1007/s10611-021-09975-w

**Published:** 2021-07-17

**Authors:** Alice Rizzuti

**Affiliations:** Department of Criminology and Sociology, University of Hull, Hull, UK

## Abstract

The food sector is subject to illegal practices of various types such as adulteration or exploitation of labour. In the media and public discourse, this phenomenon is often associated to activities by organised crime groups. Drawing on a socio-legal empirical study on the perception and conceptualisation of food crime in English and Italian public institutions, this paper unpacks the involvement of organised crime and mafia-type actors in the food sector. Considering data collected through in-depth interviews with representatives of law enforcement and other public authorities, supported by documentary sources, this research points out that, from both an institutional perspective that narrowly conceptualises as food crime as food fraud, as well as from a wider perspective that addresses other practices happening in the food sector, organised crime is involved in food crime. By referring to the English and Italian cases, and by merging different bodies of literature, such as green criminology and enterprise theory, this article advocates for conceptual clarity when referring to the involvement of corporate crime, organised crime and mafia-type groups active in the food sector. In so doing, it presents and reflects upon ‘organised food crime’ as a new socio-legal category and highlights its policy outcomes.

## Introduction

The food sector is constantly subject to illegal activities such as adulteration or exploitation of labour happening at global level. In addition, there are harmful or quasi-criminal practices such as addition of water and pesticides to food, which provide examples of licit but questionable and detrimental food practices. The literature conceptualises these practices as *food crime* [1, 2]. Despite important studies [2–5], criminological attention towards food crime has been scant. Only recently, scholars have started to analyse the many aspects of the food industry that can be examined under the lens of criminality and deviance [6–10]. Currently, it is possible to distinguish two main standpoints in the academic study of food crime: authors who mostly focus on food fraud (comprehended as a sub-type of food crime), on the organisational aspects of fraudulent activities in the food sector, and on the policy measures taken in the prevention of these illicit practices [9, 11–13]; and other authors who adopt critical approaches within green criminology and focus on discourses around social and environmental harms as well as on issues on social (in)justice surrounding access to food [2, 14, 15]. Moreover, in both public and academic debate, food crime has been associated to organised crime [16–23]; Smith et al. [24]. For instance, in Italy the origins of mafia have historically been linked to the production and sale of lemons since, by acting like an “industry of private protection” [25]*,* mafia used to provide protection from predation to citrus producers by acting like an intermediary between producers and exporters [26]. More recently, the label *agromafie* has contributed to create the narrative according to which food crimes are illicit practices perpetrated by criminal actors, mostly of mafia-type, active in the food sector [27–29].

With important exceptions [30], the involvement of organised crime in food crime and, broadly, in the food sector has not clearly been addressed yet. To fill this gap, by adopting a comparative approach, this study focuses on the experiences of the English and Italian jurisdictions in tackling the infiltration of organised crime in food crime. Drawing on twenty-seven qualitative interviews with institutional experts and documentary sources such as official reports and court decisions**,** this article deconstructs the institutional conceptualisations on food crime into actors and activities of food crime and investigates if harmful and criminal practices happening in the food sector are committed by organised criminals. The overarching argument is that organised crime and corporate crime actors are both involved in food crime to the extent that their conceptual boundaries are blurry and, eventually, it is possible to conceptualise both phenomena under the category of *organised food crime*. Not only does this category allow us to construct food crime as a form of both corporate and organised crime, it also enables to look at the involvement of organised crime in the food sector by shifting from narrow institutional conceptualisations of food crime, which under the food crime label mostly consider practices of food fraud, towards a broader conceptualisation of food crime*s* (emphasis on the plural). Adopting the category of *organised food crime* can lead to more focus on the corporate actors involved in harmful and criminal practices in the food sector as this conceptual instrument links the corporate and organised criminals and, eventually, pushes the remits of institutional agencies to more cooperation. This article attempts to formulate this socio-legal category and, by merging appropriate literature of corporate and organised crime studies, enterprise theory, and green criminology, reflects upon its meaning and utility highlighting the benefits that this conceptual tool could provide to food agencies, including those external to criminal justice.

## Comparative approach and methodology

This article originates from a wider socio-legal research project^1^ carried out in England^2^ and Italy on the analysis of the perceptions and conceptualisations of food crime according to official institutions. It presents the findings and analytical considerations on the infiltration of organised crime and mafia-type groups in food crime and, broadly, in the food sector. In doing so, it aligns with studies of comparative criminal justice and comparative criminology aiming to understand one jurisdiction through the comparison with the other and avoid the risk of ethnocentrism as well as the one of relativism [31–37]. The two jurisdictions have been selected for several reasons. First, at the time of writing, they both applied European food law. England embraces a common law legal system, whilst Italy belongs to a civil law legacy. In this sense, illicit phenomena such as food crime might be conceptualised and tackled differently by different legal systems. However, as food crime is often a cross-border issue, it is likely to pose similar risks and challenges to different states. Second, both countries have been exposed to several food scandals that might have shaped the way food crime is perceived [38, 39]. Third, Italy has been chosen in relation to the reputation and profitability of its agri-food sector. With more than 42 billion euros in 2018, Italy is one of the largest net-exporters of national cuisine in terms of food exports [40]; on the contrary, the UK (the jurisdiction of England belonging to the UK) is one of the largest net-importers of food in the world [41]. Fourth, comparing two countries with different cultural approaches to food, food traditions and eating habits, is extremely challenging and represents a reasonable comparison from a sociological perspective [42, 43]. Indeed, culture is a factor that can distinguish systems and is used to explain differences [44]. Lastly, enriching the analysis further, the two jurisdictions under scrutiny experience different manifestations of organised crime and, subsequently, they have adopted different legal constructions and judicial tools against organised crime. Since organised crime has historically and culturally overlapped with mafia, the involvements of mafia as a specific type of organised crime have been mostly considered in Italy.

In line with a purposive and representative sampling [45, 46, 47], twenty-seven public officers and expert representatives of an institutional level – thirteen in England and fourteen in Italy – have been interviewed between July 2017 and June 2018. In England, the following agencies have been contacted: National Food Crime Unit (NFCU), Trading Standards (TS), Environmental Health Departments, Environment, Food and Rural Affairs Committee (EFRA), experts of the Elliot Review into the Integrity and Assurance of Food Supply Networks, Crown Prosecution office, City of London Police. In Italy, the following bodies have been contacted: different departments of police force (Carabinieri NAS active in the protection of health and environment, Carabinieri NAC active in the protection of the agri-food sector, Carabinieri ROS active against organised crime and terrorism), Direzione Nazionale Antimafia (National Antimafia Prosecution Office), Guardia di Finanza (Fiscal police), Central Inspectorate for Fraud Repression and Quality Protection of the Agri-food Products and Foodstuffs (ICQRF), Osservatorio Agromafie (National Observatory on Crimes in Agriculture and the Agri-food System), Agency of Customs. The sampling has been a combination of snowballing and pure purposive technique. The first interviewees have been selected through academic networks and have provided access to other representatives of public agencies and experts. In addition, matching agencies with similar competencies and areas of responsibility in both countries, a list of authorities has been drafted to select potential participants with valuable insights [45]. With the exception of a few cases where the participant did not reply or decided not to take part in the study, this combination of techniques was equally successful. Access dynamics have been problematic from time to time, mostly depending on the agency. Persistence in recruiting participants has been crucial [48]. All the interviews have been transcribed, coded and thematically analysed through NVivo, and anonymised at the stage of writing up. Official documents published by relevant agencies have been gathered to strengthen the interviews. Through online engines (e.g. Google, Lexis Nexis, The Law Pages, DeJure) and institutional websites, reports, bulletins and parliamentary reports have been analysed together with documents provided by participants. Moreover, three legal case studies have been examined (Operation Boldo^3^ in England and Operations Arbequino and Provvidenza^4^ in Italy).

As participation to social research should be voluntary and appropriately informed [49], upon approval from the researcher’s institution of affiliation, ethics has been secured by providing an information sheet and a consent form informing the participants regarding their right to withdraw from the study. Clearly this study holds a certain degree of approximation in both the relatively small sample (which proved to be effective) and, possibly, in the data interpretation. However, it is one of the aims of comparative research to produce rough data from which it is possible to understand convergences and divergences across jurisdictions [50].

## Organised crime in food crime

The academic literature distinguishes between food crime as “*crimes that directly involve the processing, production and sale of food, as well as those that are more indirectly involved in local and global food trades*” ([2], p. 167) (e.g. engaging in regulatory non-compliance, addition of chemicals, unproven scientific manipulation of food, exploitation of labour, etc.); and food fraud as “*intentional substitution, addition, tampering or misrepresentation of food, food ingredients or food packaging; or false or misleading statements made about a product for economic gain*” ([51], p. 158) (e.g. counterfeiting, watering down, mislabelling, etc.). Under the category of food fraud, some authors also include economic motivated adulteration as “*the fraudulent, intentional substitution or addition of a substance in a product for the purpose of increasing the apparent value of the product or reducing the cost of its production*” (FDA 2009, cited in Spink & Moyer [51], p. R157). Data show that in the English and Italian institutional approaches this distinction is blurry as conceptualisations of food crime overlap with food fraud. By protecting public interests such as public health and national economy, food crime is as a more nuanced form of food fraud. Briefly, in England food crime is policy-framed as a serious (and organised) food fraud, and in Italy, through a normative approach, food crime matches the criminal offences tackling food fraud. Moreover, in the criminological literature organised crime has been analysed as 1) serious and organised criminal practices mostly carried out for economic profits (e.g. drug trafficking or extortion), and 2) as a set of durable and stable illegal organisations or networks whose members systematically engage in crime [52–64]. By organised crime this article refers to an umbrella concept that refers to perspectives encapsulating illegal practices and also provision of illegal commodities for illegal profits, eventually committed by organised crime groups.

By looking at both criminal practices and criminal actors, the phenomena of food crime and organised crime can be conceptually linked: the organised nature of food crime activities as well as its institutional definitions shape food crime as a form of organised crime (*activity perspective*) in which there are relevant involvements of organised crime groups (*actor perspective).*

### The organisation of food crime activities

Through different conceptual pathways that refer to the seriousness of food fraud (England) or to what is criminalised by the criminal law (Italy), both jurisdictions conceptualise food crime as an organised food fraud by looking at fraudulent activities perpetrated in the food sector and the ways these activities are perpetrated (*activity perspective*). In England, food crime is formulated as a serious and organised food fraud that endangers food safety and authenticity (British Standards Institution et al. [65–67]). The NFCU argues that food crime is “*serious and complex food fraud”* ([67], p. 9) and refers to a series of practices from “*random acts of dishonesty by individual rogues to organised fraudulent activity by groups who knowingly set out to deceive consumers or expose them to harm*” ([68], p. 7).^5^ Accordingly, the NFCU officer emphasises that “*food crime is about serious and organised criminality undertaken by people already in the food sector.”* To attract policy resources, the Elliot Review published in the afterwards of the horse meat scandal^6^ builds upon the idea that food crime is “*an organised activity perpetrated to deceive, and or injure, those purchasing a food product”* ([66], p. 11). Moreover, one of the authors of the Review refers to the horse meat scandal as “*a case with clear evidence of organised criminal activity”.* In this approach, the feature of seriousness – as well as the organisation of the criminal/fraudulent activity – performs the essential function of upgrading food fraud to the category of food crime. In other words, the level of seriousness and organisation of the activity are the factors that distinguish food crime from food fraud: if a food-related misconduct results in a serious food fraud, then it is organised and sophisticated enough to be constructed as food crime.

The feature of seriousness has been analysed in relation to regulatory breaches against consumers such as food offences [69]. Moreover, literature and policy debates have constructed organised crime as a cluster of complex and serious crimes through the “*paradigm of seriousness*” [70, 71]. This paradigm implies that if a criminal practice is serious – if it creates severe consequences to the victims or it is punished with high penalties – then this practice is labelled and tackled as organised crime to protect national security. Conceptualising a criminal behaviour that, through the paradigm of seriousness, becomes organised seems to be applicable to the case of serious food fraud that, when upgraded to food crime, is constructed as threat against public health (food safety) and national economy (food authenticity and food quality) that, ultimately, are political interests relating to national security. In fact, referring to people’s health and country’s economic stability, they create legitimacy and consensus for government and public institutions.

In Italy, regulatory agencies and criminal justice authorities embrace a narrow conceptualisation of food crime that, by looking at the body of the law, overlaps with food fraud as criminal practice perpetrated in an organised way. The ICQRF representative argues that “*from a legal perspective, the definition of food crime coincides with food fraud.”* Similarly, the Customs officer claims that “*food crimes indicate criminally relevant violations of the offences of fraud in the penal code*.” Put differently, since the law only regulates different forms of food fraud, the conceptualisation of food crime de facto overlaps with food fraud. Furthermore, food frauds are sophisticated, organised and usually perpetrated through entrepreneurially-organised activities [72]. When organised, food fraud practices are more serious and, therefore, punished with higher penalties. In the penal code (e.g. article 474ter on the aggravating circumstances in the trade of counterfeit products), counterfeiting is charged with more severe penalties if organised and committed in a systematic way. Without explicitly framing food crime as serious food fraud, in the Italian approach there is trace of the feature of seriousness in relation to organised food frauds. When discussing the potential infiltrations of mafia-type groups in food crime, the National Antimafia Prosecution Agency argues that *“in relation to article 517quater of the penal code that regulates the trade of counterfeit products of protected designation of origins and protected geographical indication, the law does not provide higher penalties for organised activities that should be tackled specifically in relation to their seriousness”* ([73], p.3)*.* Since organised food frauds are framed yet not prosecuted as serious, the current regulation has been criticised for not appropriately addressing the organised nature of food fraud [74]. In 2015, a special parliamentary commission formulated a draft law to introduce the charge of “*agropiracy”* to fight fraudulent systematic practices in the agri-food sector. Considering the different level of seriousness of the offence, this draft (not approved by the Parliament yet) aims to criminalise food frauds committed in a systematic way by complex and organised food businesses. Additionally, it encapsulates serious and organised activities perpetrated by organised groups in cases where the organisation of such activities is not stable enough to continue in time and not perpetrated through violence, not enabling to apply the charges of simple criminal association or mafia-type association [75].

Interestingly, this activity-driven conceptualising and upgrading food crime as criminal category and for policy purposes, recalls the conceptualisation of organised crime as a cluster of serious criminal practices carried out for economic profits (e.g. drug trafficking or extortion) [55, 57, 59, 60, 62, 76]. Historically, in the US where the concept of organised crime was first formulated [77] investigations focussed on organised crime’s illegal activities (e.g. gambling or loansharking) rather than on the organisation or structure of organised crime [78, 79]. Typically, the UK has focussed on the criminal activities such as sale of drugs as essential manifestations of organised crime as professional criminals [80, 81] and, in the policy framework, organised crime is treated a national security threat that affect individuals, businesses and national economy. Sergi [76, 82] identifies one policing model – “*activity model”* – according to which, in the UK criminal justice system, organised crime is conceptualised as organised crime*s* that raise public concerns because, through a process of securitisation that relates to their seriousness, they threat national security. This conceptual perspective that pinpoints the activities as the backbone of the conceptualisation of organised crime (also for policing purposes) can be transferred to the way food crime is constructed as serious and organised food fraud in both English and Italian jurisdictions.

### Involvements of organised crime in food crime

Data show that food criminals are mainly food business or corporate actors. The NFCU argues that food crime is committed by *“food people*” and “*there is no clear evidence that organised crime has infiltrated the sector.*” Similarly, the Italian National Antimafia Prosecutor stresses that “*food criminality is a non-mafia-type organised crime with typical characters of economic or business crime usually perpetrated by agri-food criminal centres.”* Nevertheless, data also show that organised crime groups are involved in food crime in both jurisdictions. From an *actor perspective*, it can be argued that 1) corporate food actors committing food crime are usually tackled as organised criminal actors and 2) in their turn, to commit food crime, organised crime actors such as mafia-type groups act as legitimate corporate actors.

In England, the NFCU official website states that “*food crime can range from isolated acts of dishonesty by individual offenders to organised illegal activity co-ordinated by criminal networks”.* Without specifying their structure and aim, the NFCU refers to criminal networks as coordinators of activities committed in an organised system [67]. One the authors of the Elliott Review claims that “*in the horse meat scandal there was evidence of organised criminal activity*” and that “*food crime is committed by criminals who get organised and work within networks established at both national and international levels*”*.* Indeed, Operation Boldo confirms the participation of business actors such as food processors and slaughterhouses. Both the Crown Prosecutor and police detective argue that in this operation there was no clear evidence of involvement of organised crime. Yet, despite being legitimate corporate actors, the criminals of the horse meat scandal share similarities with organised crime groups or organised networks. In fact, the defendants were convicted under the offence of conspiracy to defraud, which refers to the complicity to commit a serious crime and is usually applied to tackle cross-border organised crime networks by punishing the “*agreement where two or more people agree to carry their criminal scheme into effect*” [83]. Furthermore, not only have the defendants been charged under an offence typically employed to prosecute organised crime, but they have also been convicted with confiscation of assets, a measure that hits the proceeds of crime and is applied against organised criminal networks [84]. Hence, despite being food businesses, these criminal actors have been prosecuted as organised crime actors. Even if organised crime groups are associated to illicit actors that provide illicit goods (e.g. drug) in the illegal underworld, the case of food crime shows that there are also legitimate organised conspirators committing criminal acts in the legal economy that are prosecuted and sentenced as organised crime actors. Furthermore, authorities have acknowledged the potential interest of organised crime towards food fraud [85].

In Italy, by broadly pointing at criminal activities committed in the food sector (e.g. adulteration, exploitation of labour, etc.), the label *agromafie* used in public debate implies the involvement of mafia-like actors [19, 27, 86]. However, according to the institutional conceptualisation of food crime as a serious food fraud, food crime is mostly perpetrated by corporate players [72]. The National Antimafia Prosecutor argues that food criminality is committed by “*corporate criminal actors that dress up as legitimate entrepreneurs making food crime more a business crime rather than a mafia-like crime*.” He continues by highlighting that “*regarding the legal requirements needed to apply the charge of membership in mafia-type association, food crime investigations usually do not find elements such as the use of violence or the power of intimidation*.” Likewise, as argued by the officer from the police task force against organised crime, “*mafias are not active in food frauds as they do not have the necessary know-how to commit sophisticated frauds*.” Referring to Operation Provvidenza,^7^ pointing out the business nature of the criminal activities, the National Antimafia Prosecutor affirms that “*the mafia clan involved was imitating criminal systems that are initiated by non-mafia criminal actors such as business companies.”* Framing food crime as a business crime can also mean that, to reinvest money in the legitimate food market, mafia-linked companies might use intermediaries and brokers who formally are legitimate economic actors with a specific know-how and more precise knowledge of the complexities of the food sector. Indeed, in Operation Provvidenza, through several mafia-affiliated legitimate companies, the ‘*ndrangheta* clan Piromalli was involved in olive-oil adulteration and later charged under commercial fraud and membership in mafia-type criminal association. In addition, in Operation Abequino,^8^ legitimate olive oil producers were charged under membership in unlawful association, which is a criminal offence that tackles non-mafia-type organised crime groups and requires an associative bond, an organised structure and a criminal purpose.

All in all, data show that not only mafia-type groups have been involved in food crime but, more interestingly, that corporate actors are judicially treated as organised crime actors and, vice versa, organised crime actors are policed as organised conspirators. According to this, the presence of organised criminality in the food supply chain – however labelled – is clearly relevant.

## Organised crime and mafia-type groups’ infiltrations in the food sector

Matching the public perception on the presence of organised crime in the food sector [22], institutional experts agree that organised crime groups (also of mafia-type) are involved in practices happening throughout the food supply chain such as illicit competition in food transport or money laundering in restaurants and other food services.

In England, public authorities identify the food supply chain as a vehicle for the commission of criminal activities beyond food crime as it is institutionally defined. The NFCU mentions links between “*food businesses and organised crime groups whose main activity is not in itself food crime”* ([67], p. 5). Through food business models and food system structures, organised crime groups (also of mafia-type) and their affiliates use restaurants to commit money laundering [87, 88] or cover the importation of drugs inside food cargo. Due to historical and cultural biases, the narrative on Italian mafias is not integrated in the English institutional narrative on organised crime that focuses on serious and organised crime*s*. This approach could explain why mafia-type groups are usually excluded from institutional narratives on food crime in England. One of the interviewees argues that “*money laundering and drug trafficking are organised crimes more serious than food crime.*” From a law enforcement perspective, since policing these forms of serious criminality is usually prioritised, associating crimes such as money laundering committed in the food industry with the category of food crime (adopting a broader perspective on food crime) might help to increase policing resources in the food sector and identify further potential food crimes.

In Italy, institutions argue that food frauds are perpetrated by non-mafia-type organised crime actors [72]. Nonetheless, they also acknowledge mafia infiltration in the food sector in relation to typical mafia-type offences such as extortion, money laundering and illicit competition perpetrated through violence or intimidation. For example, the National Antimafia Prosecution Office identifies several mafia-like infiltrations happening inside the agri-food sector at the different stages of the food supply chain such as logistics, transport and distribution of food products [73]. Moreover, the expert from Customs argues that “*organised crime is active in the food sector in relation to loan services or in the management of fruit and vegetable markets (…) They benefit from the sale and from the logistics.”* Indeed, there have been cases of disruption in the market competition of food logistics to benefit companies belonging to mafia groups [89] and in the wholesale with mafia-type groups controlling entire vegetable and fruit markets [90]. To provide some examples, in Operation Acero-Krupi^9^ mafia-type groups have used food trucks and canned food to hide and transport drugs and weapons [91]; in Operation Pollino,^10^ mafia clans used restaurants and other food catering services for money laundering purposes; lastly, Operation Nebrodi^11^ has unveiled infiltration of mafias in EU farm subsidies frauds [92].

The final aim of organised crime groups infiltrating the food sector appears wide-ranging. Certainly, the food market is economically profitable [85, 93]. Moreover, food is a relevant resource through which organised crime, especially of mafia-type, can establish businesses and control new markets and territories [94]. Scant checks at the start of the supply chain, scarce level of investigative resources (e.g. local authorities in England), soft and low-deterrent penalties and light fiscal requirements (e.g. in the agri-food in Italy) might increase the attractiveness of the food sector in the eyes of organised crime groups. Additionally, economic sectors like food and agriculture are more vulnerable to mafia infiltration as they are less technologically-driven and predominantly based on local small-scale competition [95]. Last, in times of economic crisis like the one posed by the Covid-19 pandemic, organised crime groups can provide quick financial resources to legitimate businesses by schemes of loansharking or money laundering.

## The socio-legal category of *organised food crime*

This study has conducted a conceptual exercise upon the formulation of the socio-legal category of *organised food crime*, reflecting upon its definition, meaning and convenience in the practical fight against food crime*s* (emphasis on the plural). As food crime tends to be perpetrated through criminal networks, this section discusses if food crime networks of both activities and actors can be typified and classified in criminological terms under the category of *organised food crime*. The backbone argument is that food crime can be framed as an organised form of business crime where there are involvements of organised criminal actors acting like legitimate economic actors and involvements of legitimate corporate actors acting like organised crime. This category aims to address the whole spectrum of illicit activities happening in the food sector (e.g. food fraud and exploitation of labour as well as misleading yet legal packaging practices or legal use of chemicals) committed by corporate and organised crime actors. Here the three dimensions discussed above – activity perspective and actor perspective in food crime and involvements of organised crime and mafia-type groups in the food sector – are merged. Different bodies of criminological literature such as corporate crime theories, organised crime studies and green criminology are needed to support the conceptual construction of *organised food crime* on the basis of the experiences of national law enforcement agencies and other food institutions active in the fight against offences in the food sector. *Organised food crime* suggests a conceptual tool and identifies corresponding legal instruments to help tackling the actual involvement of both organised and corporate crime in the food market.

As seen, data show that from an institutional perspective food crime is a serious corporate food fraud. In other words, it can be categorised as an economic crime with (little) presence of organised crime and high involvement of corporate actors. From an activity perspective, food crime is organised and, because of its seriousness, falls under the category of organised crime (conceptualised as a set of serious criminal acts); from an actor perspective, there is evidence of organised crime (also of mafia-type) involvement in food crime (see Operation Provvidenza) and, additionally, corporate food actors are prosecuted and sentenced as organised criminals (see Operation Boldo and Operation Arbequino). Furthermore, organised crime groups (also of mafia-type) are highly involved in food-related activities such as money laundering in food catering or drug trafficking in food transport and logistics. It can be argued that: a) from both activity and actor perspectives, the concept of food crime is constructed as a set of organised activities and structures or actors [52, 55–59, 62–64, 82, 96]; b) in food crime, the borders between corporate and organised crime are blurry and the corresponding theoretical conceptualisations seem to overlap [97]; c) by expanding the institutional perception of food crime towards an all-encompassing concept that covers a broader range of harmful and criminal activities happening in the food sector, several forms of organised crime and mafia-type infiltration can be detected. This is supported by a green criminological perspective that enables to expand the institutional, legalistic conceptualisations to include food harms beyond legal definitions [2, 5, 14, 15, 98, 99]. Moreover, the conceptual similarity between food crime and organised crime, and the blurry boundaries between organised and corporate crime in food crime, are supported by theories of corporate and organised crime. Last, the category of *organised food crime* is constructed by drawing on the theory of enterprise formulated by Dwight Smith [79, 100].

The conceptual edges among white-collar crime, corporate crime, fraud and organised crime as well as their similarities and points of intersections have often been scrutinised [81, 97, 101–103]. Ruggiero [97] argues that these phenomena must be analysed jointly as a clear difference between corporate and organised crime is difficult to make. The conceptual blurriness between organised and corporate crime is very visible in issues of food crime. According to Croall [1], food offences cross different areas of white-collar, corporate and organised crime with a wide category of criminal actors involved (e.g. corporations involved in food manufacture, distribution and retailing, smaller businesses, gang-masters, opportunistic entrepreneurs). The scholar argues that “*food crime demonstrates the limitations of fixed categories as it also involves more traditional organised criminals and provides an example of how both legitimate and illegitimate industries may collude or how organised crime may provide a service to legitimate industry”* ([1], p. 224). Certainly, the globalisation of food systems has increased the similarity between legitimate enterprises and organised crime groups active in the food marketplace [80, 104]. On the one hand, organised crime is interested in legitimate economies such as food and acts like legitimate business-syndicates by sharing the same organisational models and structures [53, 54, 77, 103, 105, 106]. In this perspective, organised crime is framed as persistent clusters of firms with the internal organisation of a large enterprise and the aim of supplying (both legal and illegal) goods and services in order to control the market and make profits [107–110]. On the other hand, legitimate economic actors are attracted by criminal opportunities and act unlawfully in highly criminogenic sectors such as food in order to boost their profits [9, 10]. Indeed, the dysfunctionalities of the food system are ideal for corporate crime phenomena to arise: as argued by Croall [101], food offences can be typified as organisational crimes and harms committed by corporations and business companies that behave criminally when performing illegal, unethical and immoral practices in search of profits.

Criminal organisations committing cross-border crimes are like transnational corporations for the structure and scope of their operations [111]. In food crime, arguments on the transnational dimension of criminal activities committed by corporate actors and organised crime groups are supported by legal case evidence. For instance, as seen in Operations Boldo and Arbequino, legitimate food companies (meat slaughters and olive oil producers) were operating like cross-border criminal networks. Additionally, Not only does corporate crime share structural and behavioural similarities with organised crime to the extent that their respective conceptualisations are often considered in joint analysis, but corporate actors are also legally charged as organised crime phenomena (conspiracy to defraud and membership in unlawful association).

The concept of *organised food crime* represents a first attempt to mature the current conceptual and policy construction of food crime, aiming to pose more attention to a wider category of food crimes and harms and to the actual criminal actors involved. In this conceptualisation, organised crime in food crime essentially unfolds as professional crime, which is how organised crime has historically been conceptualised in the UK [80, 81]. Put differently, in this view, *organised food crime* is serious, professional crime committed by a large spectrum of actors from purely illegal organised crime groups (also of mafia-type) to legitimate business actors performing illegal acts. As mentioned, by focusing on the organisation of serious criminal behaviours in food crime and how factors of the food market influence criminals’ motivations, the enterprise theory [79, 100] offers the ideal theoretical ground to support the category of *organised food crime* and it has already been used in the study of food fraud [112]. Suggesting a paradigm shift that overtakes the alien conspiracy theory [54], in the enterprise theory organised crime is constructed as a form of enterprise that exists along the business spectrum due to the demand for certain goods, low risk of detection, low deterrence, and high profits. By considering the similarities between organised crime and white-collar or corporate crime along this spectrum, for Smith “*an illicit enterprise is the extension of legitimate market activities into areas which are normally proscribed for the pursuit of profit and in response to latent illicit demand*” ([100], p. 336). Interestingly, this approach has been applied by Passas and Nelken [113] to the study of European Community subsidy frauds perpetrated by legal and illegal firms since the authors argued that in this case the traditional conceptual separation between organised crime and white-collar crime was not applicable. Through this theoretical lens, it becomes possible to consider the whole range of illegitimate practices taking place across the food sector and include both legitimate economic actors (acting illegally) and organised crime actors (performing as business actors) without the need to refer exclusively to organised or corporate crime.

Clearly, *organised food crime* could pose conceptual and terminological unclarity. However, by expanding the organised crime label to include professional food crime would be highly beneficial from a series of perspectives such as upgraded resources or increased investigative tools. Furthermore, this is probably the path that policy-makers and law enforcement are already heading to. For instance, in preparation of the second phase of its development, the UK NFCU set to operate in line with the government’s Serious and Organised Crime Strategy through the adoption of the so-called ‘4P’ approach (preparing, preventing, pursuing and protecting) that is used to tackle organised crime [114].

## Conclusions and policy outcomes

This article has unpacked the involvements of organised crime in food crime according to the perception and reaction of relevant institutions of the English and Italian jurisdictions. It has shown that, from both institutional perspectives that focus on food crime as serious and organised food fraud as well as from a perspective that includes other practices happening in the food sector (e.g. exploitation of labour, illegal pricing, etc.), food crime is committed by organised crime as well as by corporate crime. Furthermore, since food crime is a form of economic or business crime, the conceptual edges between corporate and organised crime are unclear. The study has adopted an activity perspective to argue that, from an institutional side, food crime is conceptualised as an organised crime activity. By embracing an actor perspective, it has focussed on the involvement of corporate actors prosecuted as organised crime networks and the involvement of organised crime (also of mafia-type) groups acting like legitimate economic actors. To clarify, even within different criminal justice legal systems, if a member or affiliated to an organised crime group (also of mafia-type) committed a food crime practice with the aim of profiting the criminal group, this could also fall under the *organised food crime* category (from an actor perspective). Moreover, looking at how food crime is serious and organised (from an activity perspective) enables to consider as a form of *organised food crime* the practice (e.g. fraud) committed by a member or affiliated to an organised crime groups (also of mafia-type) in the context of a private food business not related to the purposes or proceeds of the criminal group of affiliation. Furthermore, the article has highlighted the presence of organised crime in the food sector in relation to food-related practices such as money laundering in food service or the special use of food containers to hide drugs. Finally, by referring to green criminological perspectives on food crime and to a theoretical interpretation that positions organised and corporate crime in the enterprise spectrum, this study has attempted to construct the conceptual socio-legal category of *organised food crime*. This category could provide important policy outcomes in terms of conceptualisation, policing, prosecuting, and sentencing food crime.

First and foremost, in relation to the food crime activities, a broader view that includes reflections on food harmful (and criminal) practices articulated through green criminological lenses is extremely beneficial. Such an approach in fact enables to consider interests beyond market stability and public health such as food security, food sustainability, labour conditions in the food sector and environmental impact of the food system. This conceptual stretching appears more urgent than ever as current times are characterised by socio-economic instability triggered by events that can increase criminal opportunities. For instance, following Brexit it is still not entirely clear if there might be gaps in the UK food safety legislation. Likewise, global food systems have been at risk under the Covid-19 pandemic, which has caused economic shocks with stronger impacts on medium and small agri-food producers that, under the need of cash flow and financial liquidity, might recur to illegal loans provided by organised criminals. Additionally, this perspective moves on from the traditional frame of food offences as crimes against consumers towards a wider approach capable of highlighting the social harms caused by food crime practices, labelling victims of food crimes as food citizens rather than only consumers and, finally, considering interests such as the defence of food culture, food democracy and food sovereignty [2, 4, 5, 7, 14, 15, 98, 99, 115–118].

Second, from an operational perspective, detecting food crime by controlling the entire food supply chain could function as an instrument to detect other ‘more serious’ crimes (e.g. money laundering or labour exploitation). At the same time, other forms of ‘minor violations’ such as fiscal irregularities or torts committed in the food sector could allow law enforcement agencies to detect food crimes in a more traditional sense.

Third, framing food crime as a matter of both corporate crime and organised crime would entail conceptualising it as crime that is serious and wrong enough to justify the monetary and human resources that are currently lacking in jurisdictions such as England. A more precise acknowledgement of the involvement of organised crime in food crime could unfold more law enforcement possibilities. It could support an enlargement of the investigative tool-box typically adopted in food crime investigations to include investigative instruments that, by law, are usually available only for organised crime operations such as environmental or phone wiretapping, often necessary to detect high-technology fraudulent practices or modern slavery.

Lastly, considering the different legal legacies and cultures, a common legal tool shaped around the concept of *organised food crime* is not feasible and possibly not necessary. In fact, the experiences of England and Italy do not suggest institutions should introduce new legal charges as they already employ appropriate legal infrastructures for cases of *organised food crime*. As evidenced through the legal case studies, beyond the food regulatory framework, both jurisdictions can count on appropriate legal offences to apply to this socio-legal category and tackle organised/corporate crime in food crime (i.e. conspiracy to defraud in England and membership in unlawful association, also of mafia-type, in Italy). Yet, the adoption of a common conceptualisation of *organised food crime* based on shared conceptual grounds would allow implementation of increased cooperation amongst food regulators, cross-border police and criminal justice, which is extremely needed in the field of food crime and beyond.

## Data Availability

Not applicable.

## Footnotes

1 This publication uses data and reflections within the framework of the author’s PhD thesis.

2 Some general considerations can be applied also to the jurisdictions of Wales and Northern Ireland.

3 Operation Boldo, n. T20167392, n. T20167397, n. T20167401.

4 Operation Arbequino, Tribunale di Siena, n. 41/2012 RGNR GIP; Operation Provvidenza, Tribunale di Reggio Calabria, n. 206/2017 RGNR DDA.

5 In September 2020, the NFCU published another assessment report clarifying the approaches adopted against organised crime in the food sector.

6 The horse meat scandal is a well-known food incident that took place across Europe in 2012/13 regarding the sale of beef products adulterated with horse meat.

7 To date, this represents the only judicial case with evidenced involvement of a mafia group in food crime.

8 To date, this is the only judicial example of food fraud where the defendants have been convicted under unlawful association established to commit commercial fraud (olive oil adulteration).

9 Operation Acero-Krupi, Tribunale di Reggio Calabria, p.p. n. 7428/2010 RGNR DDA and n. 57055/12 RGNR DDA.

10 Operation Pollino-European ‘ndrangheta Connection, Tribunale di Reggio Calabria, p.p. n. 6089/2015 RGNR DDA, p.p. 2868/2016 RGNR DDA and 2109/2016 RG GIP.

11 Operation Nebrodi, Tribunale di Messina, p.p. n. 890/2016 RGNR GIP.
